# Co-designing the ‘Empower Community Health’ programme: A capacity-building initiative to support community health workers’ non-communicable disease health promotion in Ethiopia

**DOI:** 10.4102/phcfm.v18i1.5326

**Published:** 2026-07-22

**Authors:** Melaku K. Yenit, Prajwal Gyawali, Tracy L. Kolbe-Alexander

**Affiliations:** 1 School of Health, Psychological and Medical Sciences and Institute for Health Research, University of Southern Queensland, Ipswich, Australia; 2 Department of Epidemiology and Biostatistics, Institute of Public Health, College of Medicine and Health Sciences, University of Gondar, Gondar, Ethiopia; 3 School of Health, Psychological and Medical Sciences and Institute for Health Research, University of Southern Queensland, Toowoomba, Australia; 4 Manna Institute, University of Southern Queensland, Toowoomba, Australia; 5 Research Centre for Health through Physical Activity, Lifestyle and Sport, Division of Research Unit for Exercise Science and Sports Medicine, University of Cape Town, Cape Town, South Africa

**Keywords:** co-design, capacity-building, community health workers, non-communicable diseases

## Abstract

**Background:**

Non-communicable diseases (NCDs) disproportionately affect low- and middle-income countries, including Ethiopia. However, community health workers’ (CHWs’) capacity in NCD health promotion is sometimes limited.

**Aim:**

This study aimed to co-design a contextually appropriate intervention with and for CHWs to support their NCD health promotion services.

**Setting:**

This co-design study was conducted in Gondar City, northwest Ethiopia. The workshops were held in the meeting hall at the University of Gondar.

**Methods:**

A qualitative participatory action research design, guided by the PRODUCES (Problem, Objective, Design, User, Co-creators, Evaluation, and Scalability) framework, was employed. Sixteen participants, including CHWs (*n* = 6), community-dwelling adults with (*n* = 3) and without NCD diagnoses (*n* = 3), and health system managers (*n* = 4) were recruited using convenience and purposive sampling. Participants attended three workshops. In workshop 1, participants discussed the roles of CHWs in health promotion and areas for improvement. Workshop 2 focused on intervention components and delivery approaches. A draft of the intervention was reviewed during workshop 3. Discussion notes and artefacts were thematically analysed in NVivo.

**Results:**

A 6-week, multicomponent intervention, ‘Empower Community Health’, was co-designed. Key themes included: (1) knowledge capacity building, (2) personal behaviour change, and (3) community-outreach initiatives. Proposed intervention topics included: (1) learning about NCDs; (2) skill motivational interviewing; and (3) behaviour change for diet and physical activity.

**Conclusion:**

The involvement of intervention users and stakeholders allowed for the sharing of experiences and fostered ownership in the ‘Empower Community Health’ programme.

**Contribution:**

The meaningful engagement of stakeholders ensures that the intervention is tailored to their needs, fosters ownership and enhances intervention buy-in. This study provides practical guidance for future co-designed health promotion programmes.

## Introduction

Non-communicable diseases (NCDs) are the leading cause of morbidity and mortality globally. In 2021, it was responsible for an estimated 43 million deaths, equivalent to 75% of non-pandemic-related deaths and 7.3 trillion cases globally.^1^ Cardiovascular diseases account for most NCD-related deaths, causing 19 million deaths in 2021, followed by cancer (10 million deaths), chronic respiratory diseases (4 million deaths), and diabetes (over 2 million deaths).^2^ Low- and middle-income countries are disproportionately affected by NCDs, accounting for 74% of global deaths.^3,4^ The burden of NCDs in Africa has been rapidly increasing over the past two decades. NCD-related deaths in the World Health Organization (WHO) African Region increased from 24.2% of total deaths in 2000 to 37.1% in 2019. Although infectious diseases have historically been prevalent in the region’s disease burden, there is an epidemiological transition from communicable diseases to NCDs.^5^ Indeed, in Ethiopia, more than one-third of all deaths are attributed to NCDs, driven by physical inactivity, poor dietary habits, and inadequate healthcare access.^6,7^ Ethiopia, a low-income country in sub-Saharan Africa, faces structural and demographic challenges that influence healthcare delivery.^8^ These include a predominantly rural population, limited healthcare infrastructure in remote areas, workforce shortages,^9^ socioeconomic disparities, and a growing epidemiological transition from communicable to NCDs.^10,11^ In addition, population growth and increasing urbanisation further strain the health system, limiting access to preventive services and health promotion.^12^ In response to the increasing global burden of NCDs and the shortage of skilled healthcare workers, the WHO recommends a task-shifting approach, advocating for delegating selected NCD services to community health workers (CHWs) and integrating community-based strategies for effective NCD prevention.^13^

Ethiopia launched a community health programme, known as the Health Extension Program, to enhance health promotion and health care accessibility.^14,15^ The programme recruits female CHWs from local communities who have at least a secondary education to complete the 1-year health extension worker (HEW) training programme.^14^

They generally work in under-resourced environments, offering primary health care services,^16,17^ providing lifestyle education, conducting screening (e.g. hypertension) and helping individuals navigate the healthcare system. Therefore, CHWs play a role in NCD prevention and management by supporting individuals and communities in adopting healthy behaviours.^16^ However, the capacity of CHWs and their involvement in NCD health programmes could be influenced by adequate training and experience.^17,18^ Providing CHWs with training and skill development opportunities will support them to provide evidence-based care and raise community awareness of the benefits of healthy behaviours.^19^ Therefore, empowering CHWs could maximise their roles in NCD health promotion and ultimately reduce the burden of NCDs.^18,20^

Our previous research has shown that CHW capacity-building interventions are needed to enhance their knowledge and skills for community-based NCD health promotion.^17^ Studies have shown that upskilling CHWs enhances health outcomes, promotes healthier behaviours, and contributes to community well-being.^21^

Understanding the needs and preferences of those involved in NCD health services is essential to ensure greater engagement, leverage local knowledge, and foster efficient resource use when developing interventions.^22^

Although previous studies have shown the need to upskill CHWs’ capacity for NCD services, limited evidence exists on how to develop contextually tailored capacity-building interventions to support their role in NCD health promotion. Furthermore, no published studies in Ethiopia have applied participatory co-design approaches that actively engage CHWs, community members, and health system stakeholders in intervention development.

Consequently, a significant gap in knowledge remains in the use of participatory research design to develop capacity-building strategies within Ethiopia’s primary health care system. Based on this paucity of data, more research is needed to explore how to apply participatory research methods to co-design a health promotion programme that aims to support CHWs in their community-based NCD health services.

Participatory research is a collaborative method that engages end-users, consumers and stakeholders in all stages of the research process, from problem identification, intervention design, implementation and evaluation.^23,24^

Engaging with the community and stakeholders will contribute to developing a tailored health promotion programme aligned with their needs and available resources.^25,26^ The terms ‘co-creation’, ‘co-design’, and ‘co-production’ are often used interchangeably when describing initiatives involving multiple stakeholders and consumers in developing, implementing, and evaluating interventions. The term co-design, used in this study, involves active collaboration between stakeholders and consumers in designing solutions to pre-specified problems. This is different from co-creation, which is the collaborative approach of problem-solving at all stages of collaboration, from identifying the problem and defining it to generating solutions, implementation, and evaluation.^23^

**FIGURE 1:**
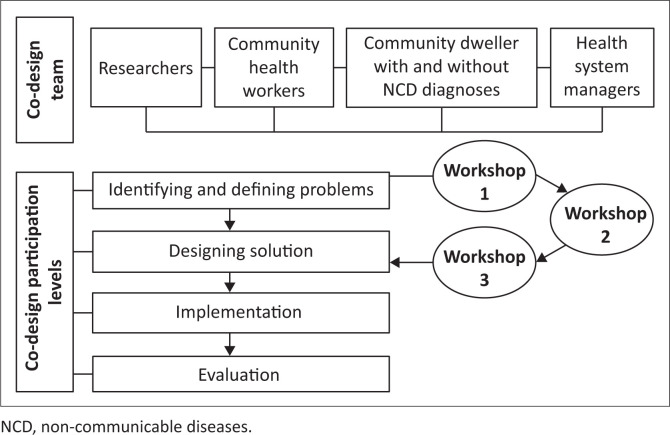
Stages of co-designing a community-based non-communicable diseases health initiative.

The aim of this research was to co-design a capacity-building intervention programme for Ethiopian CHWs in collaboration with CHWs (end-users), key health system stakeholders and community dwellers (end-consumers) (Figure 1). The study was conducted based on the PRODUCES (Problem, Objective, Design, User, Co-creators, Evaluation, and Scalability) framework, developed by the multidisciplinary Health CASCADE (https://healthcascade.eu/) expert network to provide rigorous scientific methodological guidance for participatory public health research.^27^

## Research methods and design

### Setting

The co-design workshops were conducted in Gondar City, northwest Ethiopia, between February 2024 and July 2024. Gondar City has approximately 300 000 residents and is served by approximately 150 CHWs based in one of 30 government health facilities. Workshops were held in the meeting hall at the College of Medicine and Health Sciences, University of Gondar, Ethiopia.

Ethiopia has a three-tier healthcare delivery system comprising primary, secondary, and tertiary levels. The primary level includes primary hospitals, health centres, and health posts. Health centres provide preventive, promotive, and basic curative services and oversee several affiliated health posts within their catchment areas. Health posts function as the first point of contact for community-level services and are staffed by CHWs, locally known as health extension workers (HEWs). These facilities focus primarily on health promotion, disease prevention, and selected basic curative services. Secondary and tertiary levels consist of general and specialised hospitals that provide more advanced diagnostic and clinical care.^28^

This study was conducted within the primary health care system and involved health posts and their supervising health centres. The communities served by the primary health care system include rural, urban and peri-urban populations largely dependent on agriculture, small-scale trade, and government service. Many households experience economic constraints, including low and unstable income and varying degrees of food insecurity.

Educational attainment differs across population groups, with lower levels of formal education more prevalent among older adults and rural residents. The majority of the population in Gondar City belongs to the Amhara ethnic group, and Amharic is the primary language spoken.^29^

Administratively, Gondar City is organised into 6 sub-cities and 23 kebeles, which represent the smallest administrative units.^29^ Community health workers in Ethiopia are government-employed HEWs who are recruited from the communities they serve. They are required to have completed at least secondary education and undergo formal pre-service training under the national Health Extension Program. Their responsibilities include delivering health promotion and disease prevention services, supporting maternal and child health programmes, promoting environmental sanitation, and implementing selected NCD prevention and control activities. Services are delivered primarily through health posts and community outreach, including household visits within designated catchment areas.^14^

### Study design

A qualitative participatory action research design was employed using the Health CASCADE’s PRODUCES framework. The PRODUCES framework is a structural participatory approach that supports collaborative intervention co-design through iterative engagement with users and stakeholders. Developed by the Health CASCADE expert network, it provides a rigorous scientific methodology for co-creation research in public health. The framework provides a systematic process for identifying priority topics, generating contextually relevant solutions, and refining intervention components through participants’ feedback. This framework comprises seven components: Problem, Objective, Design, User, Co-creators, Evaluation, and Scalability, to frame the study’s aim (Table 1).^27,30,31^ In this study, the framework guided the co-design process, and its application is summarised in Table 1.

**TABLE 1:** Co-designing a health promotion intervention for community health workers using the Problem, Objective, Design, User, Co-creators, Evaluation, and Scalability framework.

PRODUCES framework	Definition	Application in this study
Problem	Health issues being addressed	Informed by previous research indicating that CHWs have a suboptimal capacity and limited involvement in promoting NCD health.^17^
Objective	Aim	The goal was to co-design a capacity-building intervention to enhance CHWs’ effectiveness in promoting NCD health in their communities.
Design	Methodology used	Participatory Action Research (PAR) involved end-users and stakeholders in the co-design process.
(end-) Users	Intervention users	The co-designed intervention is intended for CHWs, support knowledge and skills acquisition for NCD health promotion.
Co-creators	Stakeholders engaged in the process	The process involved CHWs, community members (both with and without NCD diagnoses), and health system managers.
Evaluation	Evaluation of the co-design process	Measured by assessing co-designers’ satisfaction with their engagement and process relevance.
Scalability	The process of scaling an intervention	After piloting and identifying implementation barriers, the intervention can be broadly applied. This study did not include scaling up the framework’s components, as the intervention had not been piloted and barriers to implementation had not been identified.

Note: The research team adapted the PRODUCES framework from the Health CASCADE (http://healthcascade.eu/).

PRODUCES, Problem, Objective, Design, User, Co-creators, Evaluation, and Scalability; CHW, community health workers; NCD, non-communicable disease.

### Co-design processes

The co-design process was conducted in three phases, consisting of seven steps each with specific aims, activities, and deliverables (Table 2).^31^

**TABLE 2:** Processes for co-designing a capacity-building programme for Ethiopian community health workers in non-communicable disease health promotion.

Co-design phases	Co-design steps	Action taken	Team involved
Planning	Step 1: Research team-up and planning Step 2: Participant recruitment	• Literature review • Finalise methodology • Sought human research ethics • Meetings with the research team • Participants’ recruitment • Set up the co-design meeting hall	Research team
Conducting workshops	Step 3: Identification of intervention need (Workshop 1) Step 4: Intervention content development (Workshop 2) Step 5: Drafting the intervention plan Step 6: Critical review and consensus (Workshop 3)	• Participants welcomed • Conduct workshops • Established ground rules • Workshop artefacts collected	• Community health workers • Community dwellers (with and without NCD diagnoses) • Health system managers • Research team
Evaluating	Step 7: Evaluating the co-design process (Workshop 3)	• The co-design process was evaluated through a short survey	• Community health workers • Community dwellers • Health system managers • Research team

NCD, non-communicable disease.

### Planning phase

The planning phase consisted of two steps: establishing the research team and resource planning (Step 1), followed by participant recruitment (Step 2). The team undertook the literature review, drafted the rationale of the project, finalised the methodology and sought human research ethics approval. Several meetings among the research team established an understanding of the co-design process.

### Study population and participant recruitment

The study population in this co-design research is broader and includes all stakeholders actively engaged in the intervention development process. Although this study primarily aimed to develop an intervention to support CHWs, and CHWs therefore constituted the main study population, stakeholders within the community and health system were also included because of the participatory nature of the co-design approach. Therefore, the study population comprised key stakeholders directly involved in or affected by community-based NCD health promotion, who were engaged as active partners in the intervention’s co-design. A total of 16 co-design participants were recruited for the co-design workshop, consistent with the recommended 10–12 sample size for co-creation studies, which is in line with the recommended co-creator sample for participatory co-creation research.^30^ We included currently working CHWs who were available to attend three workshops:

- We recruited a diverse group of co-designers, including end-users (CHWs), community dwellers with and without NCD diagnoses, and stakeholders (health system managers). Community health workers (*n* = 6): We used convenience sampling to recruit CHWs. The managers at the Gondar Health Department sent emails to CHWs on behalf of the research team inviting them to participate. We also displayed the project advertisement on the health centre’s noticeboard, providing study information and contact details.
- Community Members (with and without NCD diagnosis) (*n* = 6): The research team contacted the community administrator at the Keble (the smallest administrative unit in Ethiopia) to promote the study to the community, in addition to a project advertisement on the noticeboard of the Keble Centre office.
- Health System Managers (*n* = 4): Similar to CHW recruitment, email invitations were sent to the health system managers. Purposive sampling was used to recruit health system managers, specifically an NCD officer, a CHW programme coordinator, and a CHW supervisor.

After potential participants expressed interest, they were provided with the Participant Information Sheet and allowed time for questions. We obtained written consent once a participant was satisfied with the information.

### Conducting co-design workshops

Three face-to-face workshops, separated by 1 week, were conducted in phase 2 and facilitated by the primary researcher. The same participants from workshop 1 were invited to participate in workshops 2 and 3. All co-design workshops were conducted in the local language (Amharic). Each workshop was organised into three main components: introduction, group activities, and consensus building. The activities undertaken during the co-design workshops are presented in Table 3.

**TABLE 3:** Co-design workshops activities.

Workshop 1	Workshop 2	Workshop 3
**Participants**		
• CHWs (*n* = 6)	• CHWs (*n* = 6)	• CHWs (*n* = 3)
• Community dwellers with NCD (*n* = 3)	• Community dwellers with NCD (*n* = 3)	• Community dwellers with NCD (*n* = 1)
• Community dwellers without NCD (*n* = 2)	• Community dwellers without NCD (*n* = 2)	• Community dwellers without NCD (*n* = 2)
• Health system manager (*n* = 3)	• Health system manager (*n* = 2)	• Health system manager (*n* = 3)
• Gender: Male (*n* = 4); Female (*n* = 10)	• Male (*n* = 4); Female (*n* = 9)	• Male (*n* = 2); Female (*n* = 7)
• Mean age (years): 34.21 (± 4.9) (min. = 25; max. = 45)		
• Mean service: CHWs = 6.7 years; health system managers = 8.3 years		
**Introduction**		
• Welcome • Brief co-design approach • Introducing workshop 1’s aim and activities • Three groups formed	• Overview workshop 1 findings • Introduce workshop 2’s aim	• Distribute the draft intervention plan • Present draft intervention plan
**Activity 1**		
CHWs’ roles and responsibilities *Aim*: To understand CHWs’ expected roles and responsibilities related to NCDs *Guide question:* • How would you describe CHWs’ role in NCD-related health promotion?	Intervention Contents *Aim:* To identify intervention components *Guide question:* • What key components should be included in the intervention?	Review and feedback *Aim:* To receive input on session 1 of the intervention draft (NCDs and Healthy lifestyles)
**Activity 2**		
NCD-related areas where CHWs are working well *Aim:* To identify and discuss the areas in which CHWs are successfully promoting NCD health *Guide question:* • What areas do you believe CHWs are working well?	Desired Outcomes *Aim:* To understand the outcomes to be achieved *Guide question:* • What outcomes should the intervention aim to achieve?	Review and feedback *Aim:* To gather input on intervention sessions related to Nutrition, meal planning, and physical activity
**Activity 3**		
Areas for improvement *Aim:* To identify NCD-related areas that need improvement *Guide question:* • What NCD-related areas do you think can be improved?	Delivery format and language *Aim:* To decide the intervention delivery format and language *Guide question:* • What is the optimal format for delivering the intervention?	Review and feedback *Aim:* To seek feedback on sessions covering behaviour change strategies, personal behaviour change, and outreach community health promotion
**Activity 4**		
-	Duration and frequency *Aim:* To decide intervention length and frequency *Guide questions:* How long and frequently should the intervention be delivered?	Review and feedback *Aim:* To receive input on the draft intervention delivery approach
**Consensus building**		
• Each group posted key discussion points on the whiteboard using sticky notes • Overall discussion • Consensus was built during each activity	• Each group posted key discussion points on the whiteboard using sticky notes • Overall discussion • Consensus was built during each activity	• Each group posted key discussion points on the whiteboard using sticky notes • Overall discussion • Consensus was built during each activity

CHWs, community health workers; max, maximum; min, minimum; NCD, non-communicable disease.

Participants were welcomed and briefed on the co-design process, the workshop’s goals and activities. Each workshop lasted approximately 2.5 h - 3.0 h, including a 20-min break. The co-design workshops were conducted using the ‘world café’ approach, a structured participatory method that facilitates collaborative dialogue through small-group, rotating discussions. In this format, participants engage in focused conversations around specific guiding questions at different tables in the same room, building on ideas generated in previous rounds and collectively refining priorities and solutions.^32^ A researcher was positioned at each table to take notes and record discussions. The workshops were designed using a world café co-design methodology, in which participants engaged in small-group discussions simultaneously within a shared space. Under these conditions, audio-recording was not feasible, as overlapping conversations would have rendered recordings difficult to attribute accurately to specific groups and challenging to transcribe meaningfully. Artefacts from the co-design workshops were generated on poster-sized sticky notes and organised using Miro software. After identifying and collecting all relevant materials, the artefacts, together with discussion notes, were thematically categorised using NVivo software (Version 14) (QSR International Pty Ltd, Massachusetts, United States [US]). A summary of workshops’ aims, guide questions and activities are presented in Table 3 and Online Appendix 1 Figure 1-OA1.

#### Workshop 1

The aim of the first workshop was to identify interventions that could empower CHWs and support them to advise on and deliver NCD health promotion initiatives. Participants were divided into three groups, each including CHWs (*n* = 2), community members (*n* = 2), and health system managers (*n* = 1). One of the participants from each group was selected as the table host, who was supported by a member of the research team. The responses were brainstormed individually, discussed as a group, and summarised on sticky notes. The participants prioritised the NCD-related content to enhance CHWs’ capacity through consensus. Key intervention areas were grouped into capacity-building, skill improvement and behaviour change.

#### Workshop 2

The objective of the second workshop was to co-design the content of the intervention based. Prior to commencing workshop 2 activities, a summary of workshop 1 was presented. Workshop 2 followed a similar structure to that of workshop 1 (Table 3). Discussions were focused on the content of the intervention, desired outcomes, delivery format and language, and the duration and frequency of the intervention.

#### Workshop 3

The aim of workshop 3 was to review the draft intervention programme. The research team drafted an intervention plan based on workshops 1 and 2, and the participants reviewed its content, applicability and feasibility. The draft programme was organised to target cognitive skill development and behaviour change techniques relevant to community-based NCD health promotion. Seven intervention sessions were proposed, covering deductive and practical learning on NCDs, nutrition and physical activity, healthy meal planning and preparation, behaviour change strategies, adapting personal behaviour change action plan, and translating knowledge into community-based outreach activities to enhance health promotion within communities. The intervention content was initially categorised into four broad areas: (1) knowledge of NCD, (2) lifestyle components (healthy diet and physical activity), (3) behaviour change and (4) application in the community.

### Data collection

Data were collected through three participatory co-design workshops. The workshops were conducted as group-based qualitative discussions rather than individual interviews. This is in line with other co-design studies. An open-ended workshop facilitation guide, developed by the research team and informed by the PRODUCES framework, was used to structure discussions and participatory activities. Data sources included artefacts generated during the workshops, such as written responses on sticky notes, flipchart summaries, structured workshop tasks, and detailed discussion minutes from in-depth group discussions.

### Rigour and co-design process evaluation

In this study, rigour was maintained through the application of the PRODUCES framework, developed by the Health CASCADE expert network, which provides systematic guidance for participatory co-creation research in public health. The framework outlines stages for identifying priorities, generating contextually relevant solutions, and refining intervention components through iterative participant engagement. We followed this approach to ensure the study’s transparency, credibility and trustworthiness. In addition, workshop discussions and artefacts were systematically documented and analysed to determine how participant contributions were generated, refined, and translated into intervention components.

A process evaluation was embedded within the co-design study. This co-design process was evaluated through a short survey adapted from the guide to co-creation,^33^ to assess participants’ satisfaction with the co-design approach, the relevance of discussion topics, their level of engagement in the process, the value of their input, and the applicability of the proposed intervention for improving NCD health promotion in communities (Online Appendix 1 Table 1-OA1). The evaluation was conducted at the end of workshop 3 and lasted 15 min.

### Data analysis

**FIGURE 2:**
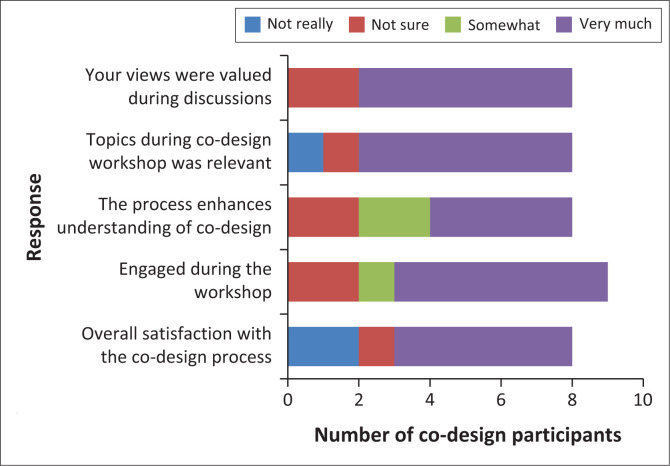
Participants’ experience of the co-design process.

Data analysis was conducted using a qualitative thematic analysis.^34^ We followed a five-step data analysis process. (1) We collected all relevant artefacts produced in each workshop; (2) Artefacts were then organised using Miro software (Online Appendix 1 Figure 2-OA1), and discussion notes were transcribed into English, and then exported to NVivo software for further analysis; (3) Using NVivo software, relevant information was extracted from notes; (4) The extracted information was analysed thematically. These themes were systematically organised into intervention outcomes and contents; (5) we reported a summary description that aligned with each workshop’s aims. Data on the experience of participants in the co-design process were analysed using descriptive frequencies.

### Ethical considerations

Ethical clearance to conduct this study was obtained from the Human Research Ethics Committee of University of Southern Queensland (approval no.: ETH2023-0851) and University of Gondar (No. CMHSH R/C/TT/06/02/57/01/2016). Workshop participants were informed about the study, its nature and procedure. Written consent was taken and participants were informed that their involvement in the workshop was voluntary and will not have negative impact on their work or health service from the health system. We ensured the privacy and confidentiality of all participants throughout the study. As there were diverse participants, we recognised the potential for unequal power dynamics in our workshop. To address this, participants were informed that their perspectives are equally valued, and respected. Furthermore, the researcher created an inclusive space for all participants to ensure equal participation.

## Results

### Participants’ characteristics

Participants’ number and characteristics are presented in Table 3. The same participants attended workshops 1, 2 and 3.

### Community health workers’ roles in non-communicable diseases health promotion and areas for improvement

Participants acknowledged that CHWs were providing health education, promotion, screening, and referral services. However, participants indicated the irregular delivery of these services and highlighted the need to empower CHWs to undertake these roles effectively via capacity-building intervention.

Participants identified five primary roles for CHWs in promoting NCD health within their communities: (1) raising NCDs and healthy lifestyle awareness (health education and promotion); (2) conducting community-based NCD screening; (3) referring individuals for diagnosis and follow-up, when necessary; (4) supporting medication adherence for community dwellers with NCD diagnoses; and (5) reporting health outcomes and performance.

### Desired outcomes from the capacity-building intervention

Four main desired outcome measures were identified for the capacity-building intervention: (1) improving CHWs’ knowledge of NCDs and healthy living; (2) developing their practical skills in meal planning and preparation, physical activity, and communication; (3) capacity for facilitating behaviour change to increase duration of physical activity and consumption of a healthy diet; and (4) application of knowledge and skill within communities. In addition, participants also indicated that CHWs could serve as role models for healthy diet and physical activity and should be included in outcome measures.

### Intervention

The final intervention programme, ‘Empower Community Health Program’, was drafted, which includes multifaceted intervention components. Participants prioritised the following intervention topics: (1) learning about NCDs, nutrition, and physical activity; (2) skill development in healthy meal preparation and motivational interviewing; (3) behaviour change for a healthy diet and physical activity; and (4) community application of knowledge and skills (Online Appendix 1 Table 2-OA1). For the delivery of the intervention, participants generally agreed that it should include both theoretical and practical components. Participants discussed the application of training within communities and finally agreed that CHWs should be first trained to deliver the programme. Participants named the intervention ‘የማህበረሰብ ጤና ማሻሻያ ስልጠና’ in the local language, translated by the research team as ‘Empower Community Health’.

A blended learning approach that combines in-person interactive sessions with practical demonstrations was preferred. In addition, interactive training sessions with case scenarios were highlighted as an effective method to encourage engagement. Participants suggested using social media platforms, such as Telegram, to enhance accessibility. Some argued that potential internet connectivity issues could challenge its implementation. Regarding the delivery language, all groups unanimously agreed to deliver the intervention in English and Amharic (the local language). The preferred intervention duration and frequency varied among groups, ranging from 4 weeks to 6 weeks. The programme was designed to be delivered over 6 weeks, with one session planned for each week (Table 4).

**TABLE 4:** Empower community health programme for community health workers’ capacity building in non-communicable disease health promotion.

Topic	Week	Session objectives
NCDs and Healthy Lifestyles	Week 1	• Understand the epidemiology of NCDs and their risk factors • Identify components of healthy lifestyles • Familiarise the WHO guidelines for healthy living
Nutrition education	Week 2	• Describe the roles of macronutrients and micronutrients • Explain dietary recommendations for a balanced diet • Identify food groups • Demonstrate portion control techniques • Nutritional assessment techniques to evaluate dietary intake
Healthy meal planning and preparation	Week 3	• Develop a healthy meal plan based on dietary guidelines • Prepare healthy meals using appropriate cooking techniques
Physical activity	Week 4	• Understand the basics of physical activity • Explore various physical activity options and techniques • Apply physical activity guidelines to daily routines
Behaviour change strategy	Week 5	• Understand different behaviour change models • Develop skills for effective behaviour change • Employing motivational interviewing techniques • Set realistic health goals for behaviour change
Personal behaviour changes action plan	Week 6	• Create a weekly meal plan incorporating healthy foods • Develop a tailored physical activity plan
Outreach community health promotion		• Organise community-based physical activity groups • Conduct healthy cooking practices with community members to promote a healthy diet

NCDs, non-communicable diseases; WHO, World Health Organization.

### Intervention programme

Each week, the programme covers a range of topics from understanding NCDs and healthy lifestyles to practical skills such as meal planning, physical activity, and behaviour change strategies.

### Process evaluation

Overall, participants were satisfied with the co-design process. Engagement levels were generally positive, and the workshop enhanced their understanding of the co-design. Most participants felt their contributions were valued; six felt their input was appreciated, while two were neutral (Figure 2).

## Discussion

This research aimed to co-design an NCD capacity-building intervention programme for Ethiopian CHWs. Through a co-design approach, we developed a capacity-building programme, called the ‘Empower Community Health Program’, that aims to enhance the roles of CHWs in NCD health promotion. Engaging end-users, consumers, and key health system stakeholders brings diverse perspectives and fosters a sense of ownership that can enhance the implementation of the intervention.^34,35^ This co-design study aligns with existing co-design literature in its engagement of diverse stakeholders and in its use of a structured, iterative participatory process.^36^ This study was also theoretically informed and guided by an established framework, consistent with other co-design studies.^37^ However, unlike studies that progressed to pilot testing,^38,39^ our co-designed intervention was not piloted because of the political situation in the study area.

Our study identified four key priority areas to maximise the roles of CHWs promoting NCDs.^13^ These priority topics included: (1) learning about NCDs, nutrition, and physical activity; (2) skill development in healthy meal preparation and motivational interviewing; (3) behaviour change for a healthy diet and physical activity; and (4) community outreach for application of knowledge and skills. These training topics align with the WHO health system capacity recommendations.^40^ Enhancing the capacities of CHWs to prevent NCDs and promote healthy lifestyles is essential for effective task-sharing and task-shifting of NCD health services.^13^ Training CHWs can empower them to become agents of change within their communities and promote healthier living. Previous studies indicate that trained CHW-led interventions have significantly improved health outcomes by increasing awareness, conducting screening and promoting healthy living.^41,42^ By focusing on capacity-building on NCDs and healthy lifestyles, we can enable CHWs to effectively address behavioural risk factors and help reduce the global burden of NCDs.^43^

This co-design study indicated that engaging with end-users, consumers and stakeholders in the design process facilitated incorporating their suggestions and preferences into the intervention. Unlike traditional, researcher-led interventions, our approach actively involved non-researcher participants in the development of the programme. Our approach was very inclusive in terms of engaging with relevant stakeholders and included input from CHWs, local residents, and the health system. Evidence shows that this inclusivity fostered a sense of ownership among participants, which could contribute to the programme’s effectiveness and sustainability.^35,44^ Moreover, our collaborative approach aligns with principles from community-based participatory research and participatory implementation science approaches, emphasising the active involvement of patients, community members, and health system representatives.^45,46^ This is in contrast to interventions that apply a top-down approach when designing and implementing interventions without involving users and stakeholders.^25^ Many researcher-led interventions are often shaped by the researchers’ perspectives and may not adequately meet the needs of the target population, resulting in limited success and sustainability, particularly in the low- and middle-income countries.^30,47^

In addition, our co-design study indicated that proper planning, fit-for-purpose participant recruitment, and active participant engagement were essential to achieving its goals. This is because proper planning and participant recruitment ensure that the right individuals are involved in the participatory research process and resources are available to create an inclusive environment and conduct meaningful engagement and collaboration. However, in the traditional research approach, participants’ selection is often guided by predefined eligibility criteria that aim primarily to ensure representation rather than collaboration. This is in contrast with the co-design research approach that prioritises recruiting participants as equal partners and knowledge contributors, rather than as subjects of study.

Furthermore, in our approach, participants contributed to identifying challenges, setting priority areas of intervention, and shaping strategies. This level of engagement enables a deeper understanding of participants’ needs and ensures that the intervention is more aligned with their experiences.^35,44^ Genuine participation involving participants throughout the intervention process, from problem identification to solution design and implementation, can lead to more tailored health strategies.^35,45^ Previous research involved participants at different levels, and the so-called ‘experts’ ultimately shaped the overall intervention plan. This approach can sometimes contradict the fundamental principle of co-design, which emphasises equal power and collaboration between participants and subject matter experts throughout the process.^48,49^

The study also provided practical guidance for future researchers seeking to co-design similar initiatives.

Successful participant engagement was a cornerstone of our approach. In this study, most participants were satisfied with the process, their engagement, the relevance of topics discussed, and the inclusiveness of their views in the intervention. The basic essence of our co-design study was the belief that *every co-design participant is an expert in their own experiences*.^50^ This assumption helped to minimise implicit power imbalance among stakeholders and promoted a sense of ownership over the programme. Furthermore, including adequate fit-for-purpose participants is key for co-design research to develop tailored interventions and replicate the process in similar settings. While a larger sample size is required for research to ensure generalisability, the number of participants in our study exceeds the recommended 10–12 co-creators for co-design methods.^30^

In our co-design study, we encountered a national security situation related to political unrest that affected the participation of the co-design team. Studies show that context is a key factor when conducting co-design research, as there is no one-size-fits-all approach. While contexts differ significantly between the Global North and South, co-design research in the low- and middle-income countries often faces more complex challenges that can impact the co-design process.^51,52^ Co-design research in the global north benefits from better infrastructure, resources, and higher literacy and digital access, while the low- and middle-income countries face challenges such as limited resources, diverse cultural norms, lower literacy, and political instability.^52^ Evidence shows that when the co-design process is tailored to the context, user and stakeholder involvement and active participation are improved.^53,54^

### Strengths and limitations

The study utilised the PRODUCES framework using the Health CASCADE network to guide the development of the capacity-building programme for CHWs. The framework is a structured participatory guideline that emphasises stakeholder engagement and is a useful tool for co-designing health interventions. We included diverse participants, collaborated to identify their intervention needs and content, and shaped the intervention programme. Although the study involved end-users, consumers, and key stakeholders, the study cannot be generalised to other countries. The intervention developed in this study may not be globally applicable; however, the process provides a framework for future low- and middle-income countries research that seeks to co-design interventions for CHWs. Furthermore, including diverse participants in our co-design process allows a wide range of views in the process.

### Implications

This study demonstrated the methodological value of using a co-design approach in developing interventions for CHWs. By actively involving intervention users, community members with and without NCDs, and health system stakeholders, the co-design process ensured that the intervention was tailored to local needs, preferences, and context. Previously, interventions for CHWs were primarily developed by researchers with limited involvement from intervention users, consumers and stakeholders. The co-designed intervention, ‘Empower Community Health’, was developed collaboratively with users, consumers and health system stakeholders to build both theoretical knowledge and practical skills for promoting healthy lifestyles and preventing and managing NCDs within Ethiopian communities. This programme has the potential to strengthen CHWs’ capacity to deliver community-based NCD health promotion and aligns with the WHO’s task-shifting strategy, which promotes the delegation of selected NCD services to CHWs. Methodologically, the study shows that co-design is a feasible and rigorous approach for creating CHW-focused interventions, offering a model for future public health research seeking to integrate experiential knowledge with evidence-based practice. Furthermore, this study provides practical guidance for researchers and practitioners seeking to co-design similar health promotion programmes tailored to local context and health system.

## Conclusion

Using the PRODUCES co-design framework,^27,31^ we co-designed a capacity-building programme, ‘Empower Community Health’ Program, to support CHWs’ capacity to deliver NCD health promotion initiatives in Ethiopia. The involvement of intervention users and stakeholders in the process allowed for the sharing of experiences and needs and fostered a sense of ownership in the programme. This is one of the first co-designed programmes for CHWs in Ethiopia to maximise their roles in NCD health promotion.

## References

[1] Li J, Pandian V, Davidson PM, Song Y, Chen N, Fong DYT. Burden and attributable risk factors of non-communicable diseases and subtypes in 204 countries and territories, 1990–2021: A systematic analysis for the global burden of disease study 2021. Int J Surg. 2025;111(3):2385–2397. 10.1097/JS9.0000000000002260 39869379 PMC12372739

[2] WHO. Key facts: Noncommunicable diseases [homepage on the Internet]. 2025 [cited 2026 Apr 27]. Available from: https://www.who.int/news-room/fact-sheets/detail/noncommunicable-diseases

[3] Allen LN, Wigley S, Holmer H. Implementation of non-communicable disease policies from 2015 to 2020: A geopolitical analysis of 194 countries. Lancet Glob Health. 2021;9(11):e1528–e1538. 10.1016/S2214-109X(21)00359-4 34678197

[4] Roth GA, Abate D, Abate KH, et al. Global, regional, and national age-sex-specific mortality for 282 causes of death in 195 countries and territories, 1980–2017: A systematic analysis for the Global Burden of Disease Study 2017. The Lancet [serial online]. 2018 [cited 2025 Mar 13];392(10159):1736–1788. Available from: https://pmc.ncbi.nlm.nih.gov/articles/PMC6227606/10.1016/S0140-6736(18)32203-7 PMC6227606 30496103

[5] Barry A, Impouma B, Wolfe CM, et al. Non-communicable diseases in the WHO African region: Analysis of risk factors, mortality, and responses based on WHO data. Sci Rep. 2025;15(1):12288. 10.1038/s41598-025-97180-3 40210980 PMC11986008

[6] Gelaw YA, Koye DN, Alene KA, et al. Socio-demographic correlates of unhealthy lifestyle in Ethiopia: A secondary analysis of a national survey. BMC Public Health. 2023;23(1):1528. 10.1186/s12889-023-16436-7 37568091 PMC10416504

[7] Kassa M, Grace J. The global burden and perspectives on non-communicable diseases (NCDs) and the prevention, data availability and systems approach of NCDs in low-resource countries. In: Anugwom EE, Awofeso N, editors. Public health in developing countries: Challenges and opportunities. London: IntechOpen; 2019.

[8] Tessema ZT, Worku MG, Tesema GA, et al. Determinants of accessing healthcare in sub-Saharan Africa: A mixed-effect analysis of recent Demographic and Health Surveys from 36 countries. BMJ Open. 2022;12(1):e054397. 10.1136/bmjopen-2021-054397 PMC8804632 35105635

[9] Hilo AA, McPeak J. Addressing concerns of access and distribution of health workforce: A discrete choice experiment to develop rural attraction and retention strategies in southwestern Ethiopia. BMC Health Serv Res. 2024;24(1):1603. 10.1186/s12913-024-11971-4 39696273 PMC11654134

[10] Tesema AG, Ajisegiri WS, Abimbola S, et al. How well are non-communicable disease services being integrated into primary health care in Africa: A review of progress against World Health Organization’s African regional targets. PLoS One. 2020;15(10):e0240984. 10.1371/journal.pone.0240984 33091037 PMC7580905

[11] Allen L, Williams J, Townsend N, et al. Socioeconomic status and non-communicable disease behavioural risk factors in low-income and lower-middle-income countries: A systematic review. Lancet Glob Health. 2017;5(3):e277–e289. 10.1016/S2214-109X(17)30058-X 28193397 PMC5673683

[12] Misganaw A, Naghavi M, Walker A, et al. Progress in health among regions of Ethiopia, 1990–2019: A subnational country analysis for the Global Burden of Disease Study 2019. The Lancet. 2022;399(10332):1322–1335. 10.1016/S0140-6736(21)02868-3 PMC8987934 35294898

[13] Joshi R, Peiris D. Task-sharing for the prevention and control of non-communicable diseases. Lancet Glob Health. 2019;7(6):e686–e687. 10.1016/S2214-109X(19)30161-5 31097265

[14] Assefa Y, Gelaw YA, Hill PS, Taye BW, Van Damme W. Community health extension program of Ethiopia, 2003–2018: Successes and challenges toward universal coverage for primary healthcare services. Glob Health. 2019;15:1–11. 10.1186/s12992-019-0470-1 PMC6434624 30914055

[15] Fetene N, Linnander E, Fekadu B, et al. The Ethiopian health extension program and variation in health systems performance: What matters? PLoS One. 2016;11(5):e0156438. 10.1371/journal.pone.0156438 27227972 PMC4882046

[16] Hartzler AL, Tuzzio L, Hsu C, and Wagner EH. Roles and functions of community health workers in primary care. Ann Fam Med. 2018;16(3):240–245. 10.1370/afm.2208 29760028 PMC5951253

[17] Yenit MK, Kolbe-Alexander TL, Gelaye KA, et al. An evaluation of community health workers’ knowledge, attitude and personal lifestyle behaviour in non-communicable disease health promotion and their association with self-efficacy and NCD-risk perception. Int J Environ Res Public Health. 2023;20(9):5642. 10.3390/ijerph20095642 37174162 PMC10178727

[18] Tesema AG, Peiris D, Abimbola S, et al. Community health extension workers’ training and supervision in Ethiopia: Exploring impact and implementation challenges for non-communicable disease service delivery. PLOS Glob Public Health. 2022;2(11):e0001160. 10.1371/journal.pgph.0001160 36962619 PMC10021836

[19] Silverman J, Krieger J, Sayre G, Nelson K. The value of community health workers in diabetes management in low-income populations: A qualitative study. J Community Health. 2018;43:842–847. 10.1007/s10900-018-0491-3 29497934

[20] Imamatsu Y, Iwata Y, Yokoyama A, Tanaka Y, Tadaka E. Empowering community health workers in Japan: Determinants of non-communicable disease prevention competency. Healthcare. 2024;12(3):297. 10.3390/healthcare12030297 38338182 PMC10855586

[21] Vedanthan R, Kamano JH, DeLong AK, et al. Community health workers improve linkage to hypertension care in Western Kenya. J Am Coll Cardiol. 2019;74(15):1897–1906. 10.1016/j.jacc.2019.08.003 31487546 PMC6788970

[22] Klingberg S, Adhikari B, Draper CE, et al. Engaging communities in non-communicable disease research and interventions in low-and middle-income countries: A realist review protocol. BMJ Open. 2021;11(7):e050632. 10.1136/bmjopen-2021-050632 PMC8296813 34290072

[23] Vargas C, Whelan J, Brimblecombe J, Allendera S. Co-creation, co-design and co-production for public health: A perspective on definitions and distinctions. Public Health Res Pract. 2022;32(2):e3222211. 10.17061/phrp3222211 35702744

[24] Fetterman DM, Rodríguez-Campos L, Zukoski AP. Collaborative, participatory, and empowerment evaluation: Stakeholder involvement approaches. New York: Guilford Publications; 2017.

[25] Majid U, Kim C, Cako A, Gagliardi AR. Engaging stakeholders in the co-development of programs or interventions using intervention mapping: A scoping review. PLoS One. 2018;13(12):e0209826. 10.1371/journal.pone.0209826 30586425 PMC6306258

[26] Jardim PTC, Dias JM, Grande AJ, et al. Co-developing a health promotion programme for indigenous youths in Brazil: A concept mapping report. PLos One. 2023;18(2):e0269653. 10.1371/journal.pone.0269653 36791063 PMC9931109

[27] Agnello DM, Balaskas G, Steiner A, Chastin S. Methods used in co-creation within the health CASCADE co-creation database and gray literature: Systematic methods overview. Interact J Med Res. 2024;13:e59772. 10.2196/59772 39527793 PMC11589503

[28] Tarekegn SM, Tadesse D, Argaw MD, et al. Capacity and performance of primary health care in Ethiopia: A novel mixed methods measurement in low-income country. BMC Prim Care. 2025;26(1):299. 10.1186/s12875-025-02988-7 41023838 PMC12481810

[29] Alene ET. Determinant factors for the expansion of informal settlement in Gondar city, Northwest Ethiopia. J Urban Manag. 2022;11(3):321–337. 10.1016/j.jum.2022.04.005

[30] Leask CF, Sandlund M, Skelton DA, et al. Framework, principles and recommendations for utilising participatory methodologies in the co-creation and evaluation of public health interventions. Res Involv Engagem. 2019;5:1–16. 10.1177/0017896917707785 30652027 PMC6327557

[31] Agnello D, Longworth D. A draft evidence-based co-creation guideline: PRODUCES+(Version 1) [homepage on the Internet]. Zenodo; 2022 [cited 2025 May 20]. Available from: https://zenodo.org/records/8379784

[32] Ravneberg BE. Co-creating and co-producing learning environments in adult education through the World Café method. Front Educ. 2024;9:1335747. 10.3389/feduc.2024.1335747

[33] Vandael K, Dewaele A, Buysse A, Westerduin S. Guide to co-creation [homepage on the Internet]. Ghent: Ghent University & Ministry of Makers; 2018 [cited 2025 Mar 24]. Available from: http://hdl.handle.net/1854/LU-8653735

[34] Leask CF, Sandlund M, Skelton DA, Chastin SF. Co-creating a tailored public health intervention to reduce older adults’ sedentary behaviour. Health Educ J. 2017;76(5):595–608. 10.1177/0017896917707785

[35] Kennedy S, Fitzgerald R, Melia R. Engaging stakeholders in the development of a national digital mental health strategy: Reflexive thematic analysis. J Med Internet Res. 2025;27:e71601. 10.2196/71601 40460420 PMC12174891

[36] Ward ME, Brún AD, Beirne D, et al. Using co-design to develop a collective leadership intervention for healthcare teams to improve safety culture. Int J Environ Res Public Health. 2018;15(6):1182. 10.3390/ijerph15061182 29874883 PMC6025638

[37] Blake HT, Davis A, Mellow ML, et al. Co-design of a digital 24-hour time-use intervention with older adults and allied health professionals. Front Digit Health. 2025;7:1544489. 10.3389/fdgth.2025.1544489 40487986 PMC12141298

[38] Laugaland KA, Akerjordet K, Frøiland CT, Aase I. Co-creating digital educational resources to enhance quality in student nurses’ clinical education in nursing homes: Report of a co-creative process. J Adv Nurs. 2023;79(10):3899–3912. 10.1111/jan.15800 37461247

[39] Singleton A, Raeside R, Partridge SR, et al. Co-designing a lifestyle-focused text message intervention for women after breast cancer treatment: Mixed methods study. J Med Internet Res. 2021;23(6):e27076. 10.2196/27076 34125072 PMC8240797

[40] WHO. Health system capacity for noncommunicable diseases management a global snapshot 2020 [homepage on the Internet]. 2020 [cited 2025 July 15]. Available from: https://www.who.int/publications/i/item/health-system-capacity-for-noncommunicable-diseases-management

[41] Teshome DF, Alemu S, Ayele TA, Atnafu A. Effect of health extension workers-led home-based multicomponent intervention on blood pressure reduction among hypertensive patients in rural districts of northwest Ethiopia: A cluster-randomised controlled trial. BMJ Open; 2024;14(8):e084029. 10.1136/bmjopen-2024-084029 PMC11344499 39181553

[42] Sankaran S, Ravi PS, Wu YE, et al. An NGO-implemented community-clinic health worker approach to providing long-term care for hypertension in a remote region of Southern India. Glob Health Sci Prac. 2017;5(4):668–677. 10.9745/GHSP-D-17-00192 PMC5752612 29284700

[43] Banatvala N, Akselrod S, Bovet P, Mendis S. The WHO global action plan for the prevention and control of NCDs 2013–2030. In: World Health Organization, editor. Noncommunicable diseases. Geneva: Routledge; 2023. p. 234–239.

[44] Maurer M, Mangrum R, Hilliard-Boone T, et al. Understanding the influence and impact of stakeholder engagement in patient-centered outcomes research: A qualitative study. J Gen Intern Med. 2022;37(Suppl 1):6–13. 10.1007/s11606-021-07104-w 35349017 PMC8993962

[45] Ramanadhan S, Davis MM, Armstrong R, et al. Participatory implementation science to increase the impact of evidence-based cancer prevention and control. Cancer Causes Control. 2018;29(3):363–369. 10.1007/s10552-018-1008-1 29417296 PMC5858707

[46] Mathieson A, Brunton L, Wilson PM. The use of patient and public involvement and engagement in the design and conduct of implementation research: A scoping review. Implement Sci Commun. 2025;6(1):42. 10.1186/s43058-025-00725-w 40211415 PMC11987400

[47] Ankomah SE, Fusheini A, Ballard C, Kumah E, Gurung G, Derrett S. Patient-public engagement strategies for health system improvement in sub-Saharan Africa: A systematic scoping review. BMC Health Serv Res. 2021;21(1):1047. 10.1186/s12913-021-07085-w 34610828 PMC8491404

[48] McKercher KA. Beyond sticky notes: Co-design for real: Mindsets, methods and movements. Australia: Beyond Sticky Notes 2020.

[49] Benz C, Scott-Jeffs W, McKercher KA, et al. Community-based participatory-research through co-design: Supporting collaboration from all sides of disability. Res Involv Engagem. 2024;10(1):47. 10.1186/s40900-024-00573-3 38730283 PMC11084036

[50] Beresford P. PPI or user involvement: Taking stock from a service user perspective in the twenty first century. Res Involv Engagem. 2020;6(1):36. 10.1186/s40900-020-00211-8 32601547 PMC7317269

[51] Mahajan SL, Ojwang L, Ahmadia GN. The good, the bad, and the ugly: Reflections on co-designing science for impact between the Global South and Global North. ICES J Mar Sci. 2023;80(2):390–393. 10.1093/icesjms/fsac115

[52] Singh DR, Sah RK, Simkhada B, Darwin Z. Potentials and challenges of using co-design in health services research in low-and middle-income countries. Glob Health Res Policy. 2023;8(1):5. 10.1186/s41256-023-00290-6 36915174 PMC10009993

[53] Peet ER, Mulder S, Mulder I. Co-design for transitions: Developing genuine participatory approaches to involving lifeworld and system participants. CoDesign. 2025:1–19. 10.1080/15710882.2025.2504456

[54] Pardoel ZE, Reijneveld SA, Postma MJ, et al. A guideline for contextual adaptation of community-based health interventions. Int J Environ Res Public Health. 2022;19(10):5790. 10.3390/ijerph19105790 35627327 PMC9141251

